# Recovery, Bioactivity, and Utilization of Bioactive Phenolic Compounds in *Citrus* Peel

**DOI:** 10.1002/fsn3.4570

**Published:** 2024-11-07

**Authors:** Nihal Durmus, Zehra Gulsunoglu‐Konuskan, Meral Kilic‐Akyilmaz

**Affiliations:** ^1^ Department of Food Engineering Istanbul Technical University Istanbul Türkiye; ^2^ Department of Food Processing Duzce University Duzce Türkiye; ^3^ Department of Nutrition and Dietetics Istanbul Aydin University Istanbul Türkiye

**Keywords:** bioactivity, *Citrus*, extraction, phenolics, upcycling, waste

## Abstract

*Citrus* peels are rich in bioactive phenolic compounds with various health effects including antioxidant, antiobesity, antiinflammatory, antihypertensive, antihypercholesterolemic, antimicrobial, antidiabetic, and anticarcinogenic activities. Both extractable and nonextractable phenolics are present in significant amounts in *Citrus* peel with diverse bioactivities. While extractable phenolics can be recovered from the fruit peels by conventional extraction methods, nonextractable phenolics remaining in the residues must be released from the cell matrix first by hydrolysis with acid, alkali, or enzymes. Novel processing technologies can help in improvement of extraction efficiency. Extreme process or medium conditions degrade phenolics and their bioactivity where encapsulation can be applied to improve their stability, solubility, and bioactivity. *Citrus* peel powder including ascorbic acid and dietary fiber besides phenolics or extracts therefrom can be used as functional food ingredients to extend shelf life and provide health benefits. In addition, phenolic extracts can be used as antioxidant and antimicrobial agents in active food packaging applications. Phenolic extracts have also a potential to be used as nutraceuticals and pharmaceuticals. In this review, phenolic compounds in different forms in *Citrus* peels, their recovery, bioactivity and possible applications for upcycling in the industry are presented.

## Graphical abstract

Citrus peels are rich in bioactive phenolic compounds present in both extractable and nonextractable fractions with various health benefits. Recovery and characterization of both phenolic fractions provide knowledge for their use. Citrus peels or extracts therefrom can be upcycled as functional ingredients or products in food, nutraceutical and pharmaceutical industries.

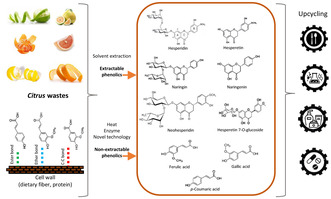

## 1 Introduction

**FIGURE 1:**
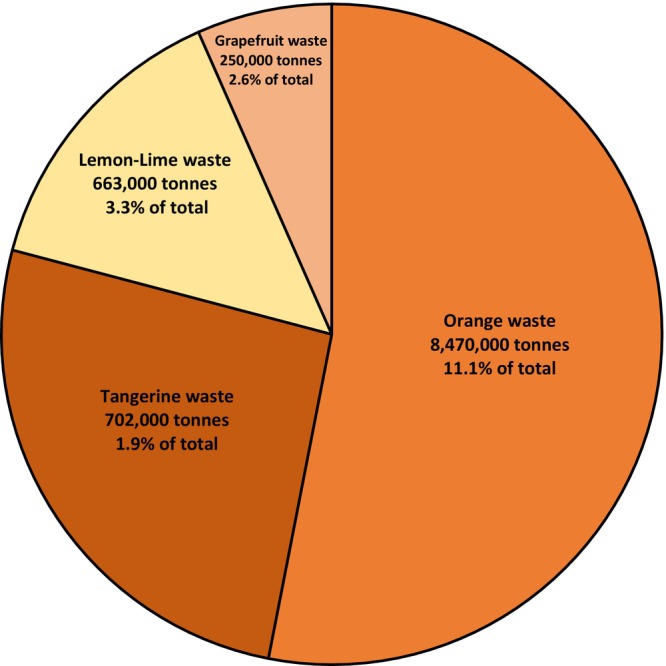
Annual production share of each *Citrus* species in the world. Estimated annual amount of waste and per cent waste in annual production for each species are noted. Amount of waste is estimated as 50% of the amount of fruit used for processing (FAO 2021).

*Citrus* fruits are part of a healthy diet with their high nutritional value, desirable sensory properties and relatively low price. *Citrus* fruits are widely cultivated and consumed fruit crops over the world due to their characteristic flavor and nutritional value being rich in vitamin C, dietary fiber and bioactive compounds. Most cultivated *Citrus* fruit varieties are orange (*Citrus sinensis*), which accounts for more than half of the *Citrus* species produced worldwide, mandarin (*C. reticulata*, *C. unshiu*, *C. tangerine*, *C. clementine*), grapefruit (*C. paradisi*), pummelo (*C. maxima* or *grandis*), lemon (*C. limon*) and lime (*C. aurantifolia*, *C. latifolia*) and they have been produced around 148 million tons worldwide in 2021 (FAOSTAT 2021). About 14% of harvested *Citrus* fruits have been utilized in the food industry for processing as juice, liquors, marmalade, jams, jellies, candy, and flavoring agents while approximately half of this amount have been discarded as waste (Figure 1) (FAO 2021).

The *Citrus* industry produces wastes mainly peel (albedo and flavedo), seed and pulp (including carpellary membranes) which constitute about 50%–70% of total fruit which are mostly used as fertilizer, animal feed, fiber or pectin source (Satari and Karimi 2018; Zayed, Badawy, and Farag 2021). The valorization of *Citrus* wastes as a renewable biological resource is becoming increasingly important for the *Citrus* processing industry to reduce the harmful impact on the environment and recycle bioactive compounds. In addition, the use of environmentally friendly methods to extract bioactive compounds from wastes is also highly valuable in today's context. *Citrus* wastes may offer a variety of bioactive constituents including pectin, dietary fiber, essential oils, organic acids, and polyphenols for the development of novel functional foods (Kaur, Panesar, and Chopra 2023a). Especially, the polyphenols present in *Citrus* by‐products have a potential to be utilized as a dietary supplement or pharmacuetical due to various health benefits. Extraction of polyphenols were identified as the most promising and reliable valorization option for a sustainable orange industry and biocatalysis was emphasized as an applicable method for the manufacture of added‐value products in a recent review by Ortiz‐Sanchez et al. (2023). Furthermore, the production of bioactive compounds with green solvents and extraction technologies have been suggested as a potential method for valorization of *Citrus* wastes (More, Jambrak, and Arya 2022).

*Citrus* wastes contain phenolic compounds particularly phenolic acids, flavonoids, and polymethoxylated flavones which display health promoting effects such as antioxidative, antihypertensive, antiinflammatory, antiobesity, anticancer, antimicrobial, hypoglycemic, and anticholesteremic activity (Mahato et al. 2018; Farag et al. 2020; Russo et al. 2021; Wang et al. 2022). The dominant phenolic compounds present in *Citrus* wastes can be different based on species, cultivar, part of the fruit, and maturity level (Wang et al. 2022; Dong et al. 2019). Although the pulp fraction contains phenolic compounds, they are present in smaller quantities compared to those in *Citrus* peel. The flavonoids in pulp fraction are mainly in the form of glycosides, whereas the peel contains a greater amount of less polar flavanone and flavone aglycons (Singh et al. 2020). *Citrus* peel, in particular, contains polymethoxylated flavones which are not commonly found in other plants.

Phenolics are present mostly in soluble or extractable form in *Citrus* peel; however, there are considerable amounts of phenolics in insoluble and bound forms which are called nonextractable phenolics (Pérez‐Jiménez, Díaz‐Rubio, and Saura‐Calixto 2014). The studies in the literature generally have focused on extractable phenolics and there are less studies about nonextractable phenolics that remain in the residues after extraction of phenolic compounds by solvents. The aim of this review is to provide an overview about phenolics present in *Citrus* peel in different forms along with their bioactivity, bioaccessibility, bioavailability, processes for their recovery and applications in various industry segments to reveal an upcycling potential.

## 2 Phenolics in *Citrus* Peel

Large amounts of solid residues (mainly peels, membranes and seeds) remain as waste as a result of processing of *Citrus* fruits. The quantity and profile of phenolic compounds in *Citrus* peel are influenced by several factors like species, variety, growth and environmental conditions, extraction methods and degree of fruit ripening. Dong et al. (2019) demonstrated that the total phenolic content (TPC) and the antioxidant activity decreased during ripening while the individual flavonoids (hesperidin, eriocitrin, diosimin, rutin, cynaroside) was found to be at a maximal level in lemon peel and pulp of fruits harvested in August. Similar findings were reported by Wang et al. (2022) for brocade orange (*C. sinensis* L. Osbeck) peel.

A wide variety of phenolic compounds are present in soluble free, soluble conjugated, and insoluble bound forms in peel of different *Citrus* species (Table 1). The contributions of bound phenolics to TPC in some *Citrus* wastes were reported to be (Oboh and Ademosun 2012) 39% in orange peel, 34% in shaddock peel, 5% in grapefruit peel, and 19% in lemon peel (Durmus and Kilic‐Akyilmaz 2023). Additionally, the glycosylated and esterified forms were found to be as 82% and 9% in orange peel albedo (Wang et al. 2022), 58% and 26% in orange peel flavedo (Wang et al. 2022), 10% and 11% in mandarin peel (Hayat et al. 2009) of TPC, respectively. Thus, a considerable portion of polyphenols remains in the residues after extraction, and causes underestimation of phenolics and antioxidant activity of *Citrus* peel.

**TABLE 1:** Literature data on TPC of various *Citrus* peel in different forms.

*Citrus* wastes	Total phenolic content (mg/g dry matter)			
	Extractable			Nonextractable
	Free	Esterified	Glycosylated	
Calamondin (*C. mitis* Blanco) peel Lou et al. (2014)	23.23–40.94	3.34	0.49	—^a^
Grapefruit (*C. paradisii*) peel Oboh and Ademosun (2012)	13.1	—	—	0.72
Lemon (*C. limon* Lamas) peel Durmus and Kilic‐Akyilmaz (2023)	16.71	—	—	3.76
Lime (*C. aurantiifolia* Swingle) peel Guimarães et al. (2010)	124.63			
Mandarin (*C. reticulata* Blanco) peel Hayat et al. (2020)	1.16–1.40	1.95–2.26	0.11–0.23	—
Orange (*C. sinensis*) peel Oboh and Ademosun (2012)	10.5	—	—	6.8
Orange (*C. sinensis* L. Osbeck) peel albedo Wang et al. (2022)	2.15–3.18	1.30–2.64	17.01–23.92	0.49–0.80
Orange (*C. sinensis* L. Osbeck) peel flavedo Wang et al. (2022)	3.16–4.59	6.30–8.68	14.25–20.17	0.89–1.09
Pummelo (*C. grandis* L. Osbeck) peel flavedo Lü et al. (2016)	6.04–11.53	—	—	—
Pummelo (*C. grandis* L. Osbeck) peel albedo Lü et al. (2016)	4.56–14.75	—	—	—
Shaddock (*C. maxima*) peel Oboh and Ademosun (2011)	6.5	—	—	3.4

^a^ Not reported.

**FIGURE 2:**
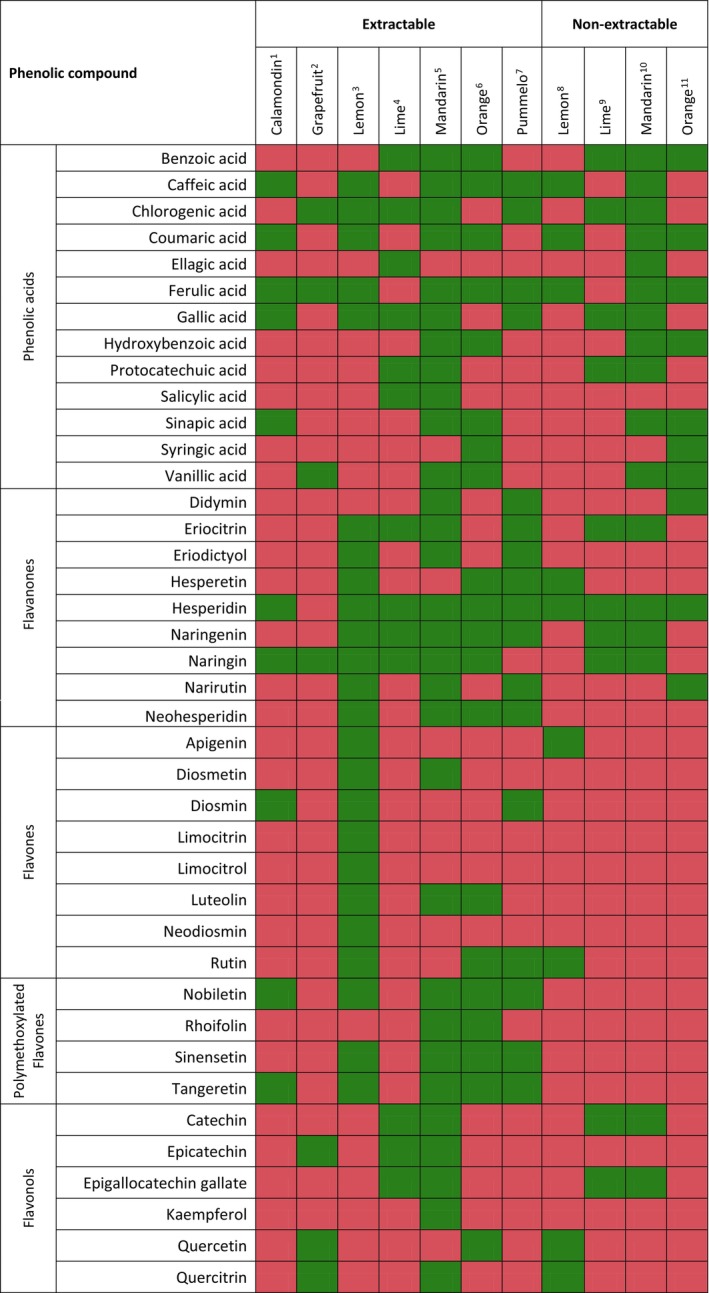
Heatmap showing distribution of phenolic compounds in different *Citrus* wastes. Red boxes mean phenolic compound does not exist; green boxes mean phenolic compound exists. ^1^Lou et al. (2014); ^2^Nishad et al. (2019); ^3^Papoutsis et al. (2017), Xi et al. (2017); ^4^Esparza‐Martínez, Miranda‐López, and Guzman‐Maldonado (2016); ^5^Esparza‐Martínez et al. (2016), Zhang et al. (2014), Hayat et al. (2020); ^6^Camacho et al. (2022), Wang et al. (2022); ^7^Lü et al. (2016); ^8^Durmus and Kilic‐Akyilmaz 2023; ^9^Esparza‐Martínez, Miranda‐López, and Guzman‐Maldonado (2016); ^10^Esparza‐Martínez et al. (2016); Zhang et al. (2014); Hayat et al. (2020); ^11^Camacho et al. (2022).

*Citrus* peel is composed of two layers, flavedo or epicarp (colored peripheral surface) and albedo or mesocarp (white soft middle layer). A wide range of phenolic compounds are present in the peel, their type and distribution mainly depend on the species (Figure 2). Phenolic acids such as caffeic, ferulic, *p*‐coumaric and sinapic acids, flavanones including narirutin, hesperidin, neohesperidin, hepseretin, eriocitrin, naringenin and naringin, flavones such as diosmetin, diosmin, apigenin and rutin and polymethoxyflavones, a unique subclass to *Citrus* species, such as nobiletin, sinensetin and tangeretin are present in *Citrus* peel (Kaur, Panesar, and Chopra 2023a). While albedo contains higher amounts of flavanones especially in glycosylated form, flavedo mostly includes flavones and flavonols, polymethoxyflavonoids and esterified forms (Aruoma et al. 2012; Wang et al. 2022). Moreover, same phenolic compounds are present in the extractable and the nonextractable fractions; however, their concentrations and form in each fraction are different. In addition, nonextractable phenolics also include condensed and hydrolyzable tannins (Nishad et al. 2019). Wang et al. (2022) reported that most of the phenolics in brocade orange peel were in glycosylated form compared to free, esterified and insoluble bound forms. Phenolic acids were found to be present more in free form compared to bound forms in orange peel (Kaur, Panesar, and Chopra 2023a; Wang et al. 2022). Hesperidin, hesperetin, and eriocitrin have also been found in both fractions (Camacho et al. 2022; Durmus and Kilic‐Akyilmaz 2023). Data from the literature indicate that *Citrus* peels are rich source of phenolic compounds present in both extractable and nonextractable fractions.

## 3 Processing Methods for the Recovery of Phenolic Compounds From *Citrus* Peel

*Citrus* peels are first dried for preservation until further processing for recovery of bioactives. Proper drying method and process parameters need to be selected for ensuring stability of bioactives. For bulk drying of *Citrus* peels, hot air drying, infrared drying or microwave drying are applicable where a temperature of 50°C–65°C can be applied for preserving bioactives (Suri et al. 2022). Heat treatment may change the profile of phenolics by degradation and/or release depending on the applied temperature and time. Wang et al. (2023) found that insoluble bound phenolics were not influenced by drying methods while glycosylated phenolics were more sensitive than esterified phenolics. Interestingly, there was no significant change in the amounts of free phenolics which was explained by the supply of them via hydrolysis of glycosylated phenolics during drying.

Extraction method used for the recovery of phenolics from *Citrus* peel primarily determines the yield and profile of phenolics (Table 2). Phenolics are commonly extracted from plant materials by maceration or conventional solvent extraction with mild heating in some cases. Soluble free or soluble conjugated phenolics can be efficiently extracted with water/organic solvents. On the other hand, nonextractable phenolics can be present as weakly bound via noncovalent bonds or physically entrapped in the cell matrix while some of these are covalently bound to cell wall structural materials such as cellulose, hemicellulose, pectin, lignin and proteins through ether, ester, and C‐C bonds (Acosta‐Estrada, Gutiérrez‐Uribe, and Serna‐Saldívar 2014; Macedo and Madeira 2020; Shahidi and Hossain 2023). Nonextractable phenolics require special treatments such as acid, base or enzyme hydrolysis for release from cellular matrix. Afterward, they are recovered from the matrix by the use of a solvent as extractable phenolics.

**TABLE 2:** Extraction methods applied for recovery of phenolics from *Citrus* peel.

Matrix	Extraction method	Results	References
Lemon by‐products (endocarp, residual membranes, and exocarp) (*C. limon*)	1 g/100 mL UAE: Ethanol (50%), 43 kHz, 150 W, 10–60 min, 30°C HWE: 15 min, 95°C CSE: Ethanol (50%), 1 h, ambient temperature	The optimum UAE parameters: 45 min, 50°C, 250 W. Same TPC, TFC and FRAP with UAE and CSE HWE yielded the highest TFC and antioxidant capacity	Papoutsis et al. (2018b)
Brocade orange peels (*C. sinensis*)	UAE: Water–methanol‐DMSO (1:4:5, v/v/v), 1 g/10–20–30 mL, 40–60–80 W, 20–40 min CSE: Water–methanol‐DMSO (1:4:5, v/v/v), m/v: 1 g/20 mL, 6 h EAE: Water with 10 U/g pectinase, cellulase, hemicellulase, and papain, 1 g/20 mL, 50°C, 6 h	Optimum parameters for UAE: Solvent/Solid ratio 17.6 mL/g, 28 min, 26°C and 60 W. UAE and EAE yielded higher amount and activity than CSE	Wang et al. (2023)
*Citrus* juice by‐products	EAE: Single or combined enzymes, 5–20 U/g cellulase, pectinase, tannase, β‐glucosidase, 6–24 h, 40°C, ethanol (50%), 2 g/25 mL, CSE: 2 g/25 mL, 15 min, 30°C	Enzymes increased the release and bio‐conversion of phenolics The highest condition for hesperetin and naringenin production was 24 h of reaction using β‐glucosidase at 20 U/g	Ruviaro et al. (2020)
Lime peel	Ethanol (50%–100%), 1.5 g/30 mL MAE: 140–700 W, 10–45 s, 60°C UAE: 43 kHz, 20%–40% amplitude, 2–4 min, 23°C–50°C	The optimum parameters for MAE: 55% ethanol, 140 W, 45 s, 8 cycles The optimum parameters for UAE: 55% ethanol, 38% amplitude, 4 min UAE was more efficient to extract the total phenolics with higher antioxidant activity and 33% time saving compared to MAE	Rodsamran and Sothornvit (2019)
Kinnow mandarin peels (*C. reticulata*) Mousambi peels (*C. limetta*)	Acetone, 1 g/3 mL UAE: 30 min, 37°C CSE: 30 min, 37°C	Slight increases in yield and antioxidant activity were obtained by UAE compared to CSE	Saini, Panesar, and Bera (2019)
Kinnow mandarin peels (*C. reticulata*)	UAE: 31% amplitude, 30:1 liquid: solid ratio, at 41°C, 15 min	Higher antioxidant activities and maximum TPC	Kaur, Panesar, and Chopra (2023b)
Mandarin peels	UAE: 400 W, 80% duty cycle, 40°C for 30 min CSE: 50% etanol, 1:10 solid:liquid ratio	UAE resulted in better recovery of TPC and TFC compared to CSE	Anticona et al. (2021)
*C. sinensis* (Malta) peel	UAE: Ethanol (50%), 1 g/20–40 mL, 43 kHz, 60%–100% amplitude, 15–35 min, room temperature EAE: Viscozyme L. at 0.7%–0.9%, 4–6 h, 60°C, ethanol (70%), 5 min, 50°C	The optimum parameters for UAE 70.89% amplitude, 40 mL/g, 35 min The optimum parameters for EAE 0.84% enzyme concentration, 30.94 mL/g, 4.87 h EAE resulted in two‐fold higher yield of phenolics than UAE	Nishad, Saha, and Kaur (2019)
Lemon flavedo (*C. limon*)	UAE: Water, 1 g: 20 mL, 40 kHz, 150 W, 20 min, room temperature (25°C) EAE: Complex enyzme with cellulase, 2 g sample with 0.1% enzyme mixture, 2 h, 50°C HHP: Water, 1 g: 20 mL, 400 MPa, 10 min, room temperature (17°C–20°C)	EAE yielded the highest TPC and α‐amylase and α‐glucosidase inhibition followed by HHP and UAE	Lu et al. (2021)

*Note:* Parameters including the solid/solvent ratio (g/mL), solvent type, frequency (kHz), power (W), amplitude (%), and time (min) are given for each method.

Abbreviations: CSE, conventional solvent extraction; EAE, enzyme‐assisted extraction; HD, hydrodistillation; HHP, high hydrostatic pressure; HWE, hot water extraction; MAE, microwave‐assisted extraction; PLE, pressurized liquid extraction; RE, rotary extraction; SFE, supercritical fluid extraction; SOX, soxhlet extraction; TPC, total phenolic content; TFC, total flavonoid content; UAE, ultrasound‐assisted extraction.

Phenolics can be recovered from plant materials by using solvents like methanol, ethanol, ethyl acetate or aqueous mixtures of these. Solvent polarity, solid: solvent ratio, particle size, temperature, and time are the main factors for optimizing the yield. Aqueous ethanol (50%–75%) was found efficient for extraction of polyphenols from various *Citrus* peels (Papoutsis et al. 2016; Manchanda et al. 2023; Chatzimitakos et al. 2023). Novel solvents such as ionic liquids and deep eutectic solvents have been applied for improving the recovery of bioactive compounds from food materials in recent years (Toprakçı, Toprakçı, and Şahin 2022). These solvents can be tailor‐made for recovery of target analyte using synthetic or natural materials. Their selectivity for certain compounds, high viscosity reducing diffusion and recyclability and high cost in the case of some solvents are major drawbacks. In addition, their scale‐up for industrial applications is required. In a recent study, Athanasiadis et al. (2024) used a surfactant, Span 20, to recover polyphenols from an aqueous extract of lemon peel by cloud point extraction where they were also encapsulated in a micelle structure. A recent review by Xu, Li et al. 2023 provides more in‐depth information on solvents for extraction of phenolics from *Citrus* waste.

Water at subcritical state with modified polarity at increased pressure (15 bar) and temperature (130°C–200°C) has been used for extraction phenolics from *Citrus* peel (Šafranko et al. 2021; Brezo‐Borjan et al. 2023). Major disadvantages of subcritical water extraction are high temperatures and long extraction times that can cause undesirable browning reactions, degradation, hydrolysis, or oxidation of phenolic compounds (Lachos‐Perez et al. 2018; Zhang et al. 2020). Supercritical carbondioxide is another green solvent that can be used at low temperatures with high extraction efficiency and no toxicity. Carbon dioxide with a low polarity is preferred for extraction of low/medium polarity or nonpolar compounds but it loses effectiveness when used to extract compounds with hydroxyl and carboxyl groups such as polar polyphenols (Gil‐Martín et al. 2022). Supercritical carbon dioxide has also a low extraction efficiency due to its inability to penetrate the complex cell wall structure (Wedamulla et al. 2022). Therefore, it may not be a good choice for the recovery of nonextractable phenolic compounds. However, its solvent properties can be useful after the hydrolysis of the cellular matrix with other treatments.

Hydrolysis of the cell matrix is required first for recovery of nonextractable phenolics. Acidic hydrolysis with hydrochloric acid or sulfuric acid breaks down the glycosidic bonds of phenolics solubilizing sugars but leaves the ester bonds intact. However, the yield maybe low as some degradation of polyphenols can occur at low pH and high temperatures (Dzah et al. 2020). Alkaline hydrolysis commonly with sodium hydroxide can break the ester bonds at low temperatures causing less degradation. However, the procedure requires special cautions for ensuring the stability of phenolics and takes a longer time (Shahidi and Hossain 2023). Thermal and nonthermal extraction methods can recover both extractable and nonextractable phenolics depending on the intensity of the treatment. Novel technologies such as ultrasound, microwave and high pressure applications are used to assist solvent extraction and increase the recovery of phenolics from plant materials. Novel technologies provide greener extractions by lowering energy consumption and use of nontoxic solvents (Putnik et al. 2017). In addition, these treatments can damage or break down cellular structure and liberate nonextractable entrapped and bound phenolics from the plant cell matrix.

Among novel technologies, ultrasound‐assisted extraction (UAE) has been used widely to extract phenolic compounds from food wastes. Low frequency ultrasound (20–40 Hz) is preferred commonly for extraction as it is more destructive on the material. However, phenolic compounds can be degraded by oxidation unless cooling is applied at the same time (Gómez‐Mejía et al. 2019). In addition, special equipment design is required for scale‐up to increase capacity and safety of workers must be ensured for efficient and safe use of this green technology (Kumar, Srivastav, and Sharanagat 2021; Perera and Alzahrani 2021). Ghasempour et al. (2019) explained the positive effect of UAE on extraction efficiency by destruction of cell matrix releasing bound phenolics, accelerated mass transfer, decrease in viscosity of the solvent and increase in solvent penetration by heat output. A particle size of 1.4 mm was found critical in UAE of aqueous *Citrus* pomace for a high yield of phenolic acids and hesperidin while an increase in temperature from 30°C to 50°C improved the recovery of hesperidin, TPC, total flavanoid content (TFC), and cupric ion reducing antioxidant capacity (CUPRAC) (Papoutsis et al. 2018a). Although the phenolic compounds present in different forms were not determined in these studies, high intensity ultrasound has the ability to homogenize food materials by application of high shear, pressure and temperature. Therefore, UAE can allow the release of physically entrapped or noncovalently bound nonextractable phenolics from the cell matrix. In a study by Durmus and Kilic‐Akyilmaz (2023), treatment of the lemon peel powder residue after extraction of soluble phenolics with high intensity ultrasound at 50% amplitude for 15 min was able to liberate some phenolic compounds especially hesperidin and hesperetin by 20% and 80%, respectively, compared to conventional heat‐assisted extraction at 40°C for 60 min. Moreover, the amounts of nonextractable phenolics obtained by UAE and conventional heat‐assisted extraction were comparable to those present in the extractable fraction.

Microwave‐assisted extraction (MAE) can also increase the extraction yield. MAE relies on fast and uniform heating of a material as a result of vibration of polar molecules trying to quickly align themselves with the microwave's high frequency electric field. Microwaves can penetrate to the interior of the plant cells or food matrix, and heat the *in situ* water throughout its volume, whereas conventional heating is from exterior and requires contact with a hot surface. As a result of heating intracellular water, pressure inside the cells increases causing swelling, rupture and hence release of the cellular contents into the solvent (Zhang et al. 2020). Quick heating also causes an increase in solubility and diffusion of target compounds into the solvent. Hayat et al. (2020) reported that choice of MAE power and heating time was important and an extension of exposure time from 10 to 15 min at 250 W was detrimental on phenolic compounds in Feutrell's Early peel. Similar to UAE, MAE has the capability of disruption of cellular structure hence aiding recovery of nonextractable phenolics. The disadvantages of MAE are thermal degradation of some phenolic compounds and high initial capital cost.

High hydrostatic pressure (HHP) is another nonthermal method applied for extraction of phenolics where high pressure between 100 and 1000 MPa is applied to a matrix via a transmitting liquid in a closed system. As a result of pressure increase, air in plant cell vacuoles leaks disrupting the cell membrane and permitting contact with the extraction solvent. In addition, HHP can disrupt noncovalent bonds between phenolics and food matrix and induce their liberation. Moreover, it can alter the conformation or denature cell membrane proteins reducing their selectivity and enhancing the diffusion of phenolics to the solvent (Ninčević Grassino et al. 2020; Cascaes Teles et al. 2020). Parameters of HHP need to be selected properly for the preserving activity of phenolics obtained from waste food materials. Heat‐sensitive phenolics can be extracted by HHP with a high yield in a shorter time. HHP‐assisted extraction of orange flavedo yielded higher antioxidant activity than conventional solvent extraction where pressures of 200 and 400 MPa were applicable, but 600 MPa reduced the antioxidant activity (Afifi et al. 2023). In the study of Afifi et al. (2023), application of pulsed electric field (PEF) at 15 kJ/kg and 3–10 kV also yielded higher antioxidant activity for orange flavedo samples than conventional solvent extraction.

High‐voltage electric discharge (HVED) is another novel technique where electric discharge is created due to application of high electric field. Electrohydraulic discharge in water causes strong shock waves in the medium, bubble formation, turbulence, emission of UV radiation, and free radicals. These events lead to cell destruction and mass transfer enhancement (Li, Fan, and Xi 2019). HVED pretreatment (222 kJ/kg) of orange peels prior to enzyme hydrolysis (ViscozymeL, 12 FBGU/g) improved extraction of polyphenols from orange peels (El‐Kantar et al. 2018). Banožić et al. (2022) also applied HVED for extraction of polyphenols from mandarin peel where optimum frequency and time were determined for extraction yield, hesperidin, and narirutin contents. This technique can also help in the recovery of nonextractable phenolics as it can degrade the cell structure.

Although many studies have shown the benefits of novel technologies on the extraction of phenolics, simple conventional solvent extraction can suffice in some cases. In addition, uncontrolled temperature increase and long duration in extraction by some novel technologies may cause reduction in phenolic content and antioxidant activity. Chatzimitakos et al. (2023) found that simple solvent extraction with 75% ethanol in water at 20°C for 150 min was found to be efficient for extraction of phenolics from lemon peel powder and application of PEF or ultrasound as a pretreatment did not change the yield. In a study by M’hiri et al. (2016), the highest antioxidant activity was obtained from Maltease orange peel by conventional extraction of phenolics at 35°C for 30 min with aqueous ethanol. It is also possible to integrate extraction of different valuable components such as pectin and phenolics if a common solvent such as water is used. Cameron, Chau, and Manthey (2016) were able to extract pectin along with phenolic compounds by steam injection at 150°C as an environmentally friendly method where 41.1% of the polymethoxylated flavones, 11.4% of the flavanone glycosides, 100% of hydroxycinnamates were recovered with a water wash. Das and Arora (2023) determined optimum conditions for obtaining hydrothermal water to be used as a one step green co‐extraction solvent for pectin and phenolic compounds from sweet lime peel as 112.2°C for 17.1 min and 14.3 mL/g liquid: solid ratio. Heat and novel techniques such as UAE, MAE and HHP can provide a simple method for recovery of extractable and some of nonextractable phenolics. However, for the complete recovery of nonextractable phenolics, acid, base, or enzymatic hydrolysis of the peel matrix is required.

Enzyme‐assisted extraction (EAE) stands out among other extraction methods because it is an environmentally friendly and safe method, does not require special equipment and preserve the structure and activity of the target compounds. The presence of a wide variety of carbohydrases capable of hydrolyzing structural carbohydrates such as pectin, cellulose, hemicellulose and arabinoxylan allows hydrolysis and extraction at mild conditions with high efficiency. These enzymes degrade cellular structure by hydrolyzing glycosidic bonds and release entrapped phenolics and those linked to the cell wall while increasing the porosity of the substrate and allowing better penetration of the solvent into the structure (Nishad, Saha, and Kaur 2019). Pectinases or pectinolytic enzymes contain more than one hydrolytic enzyme. Of these, protopectinases can solubilize insoluble protopectin as a result of the ripening process in immature fruits, while esterases or pectin methyl esterases can degrade esterified units by removing methoxy esters. Depolymerases represented by lyases and hydrolases are also involved in the breakdown of glycosidic bonds (Gligor et al. 2019). In addition, cellulases are a group of enzymes that catalyze the degradation of cellulose into cellobiose and glucose. β‐glucosidase, which is commonly used in the extraction of phenolic compounds from plant materials, is one of the enzymes covered in this group and breaks down cellobioses into glucose (Gligor et al. 2019). As there are different types of bonds in a complex matrix of plant materials, an enzyme mixture rather than a single enzyme is preferred for increasing recovery of bound phenolics. High specificity, effective action, use of water as a solvent, mild conditions of pH and temperature and short processing time are advantages of EAE. Ruviaro, Barbosa, and Macedo (2019) used pectinase, cellulase, tannase and β‐glucosidase for extraction of phenolic compounds from *Citrus* by‐products. Interestingly, single use of β‐glucosidase at 20 U/g optimally hydrolyzed phenolic compounds from sugar residues and increased antioxidant activity. Enzymatic reaction time for maximum production of hesperetin and naringenin from hesperidin and naringin was reported as 24 h.

Application of ultrasound in combination with enzyme hydrolysis has been suggested to accelerate hydrolysis reactions (Wang et al. 2017). However, in a study by Durmus and Kilic‐Akyilmaz (2023), application of both EAE and its combination with UAE for extraction of phenolics from lemon peel yielded similar concentrations of phenolic compounds, 2‐ to 4‐fold increase in concentration of individual phenolic compounds, indicating enzyme hydrolysis was solely efficient. Lu et al. (2021) also found higher phenolic content and antioxidant activity by EAE compared to those by UAE and HHP applied on lemon flavedo. Similarly, Barbosa, Ruviaro, and Macedo (2021) reported that EAE performed better than hydroalcoholic extraction and UAE in recovery of phenolics from a *Citrus* pectin by‐product. Nishad, Saha, and Kaur (2019) also obtained two‐fold higher concentrations of phenolics from *C. sinensis* cv. Malta peel by EAE with Viscozyme compared to UAE. Mushtaq et al. (2017) also recommended enzymatic hydrolysis before supercritical fluid (SCF) extraction to improve the extraction efficiency by rapid mass transfer, reduction in the particle size, increase in the contact area, enhancement of solvent distribution, and liberation of nonextractable phenolic compounds.

Fermentation‐assisted extraction of polyphenols is also possible where microorganisms produce cell wall degrading enzymes such as pectinases, lignases, cellulases, or hemicellulases. In addition to releasing bound phenolics, microbial metabolism can also transform polyphenolic compounds to simpler phenolics or other metabolites with higher bioactivity (Gulsunoglu‐Konuskan and Kilic‐Akyilmaz 2022; Vilas‐Franquesa et al. 2023). Hu et al. (2022) showed that solid‐state fermentation of *Citrus* pomace with the autochthonous probiotics *Lactobacillus plantarum* P10, M14 and *Bacillus subtilis* BF2 released phenolics by upto 133.15% by cellulase, filter paperlyase, and pectinase activities. They observed that the phenolic profile was changed after fermentation where naringin and hesperidin were the main phenolic compounds resulting in 3‐ to 4‐fold increase in free radical scavenging activity. In another study, fermentation of lime peel by *Aspergillus saitoi* for 6 days resulted in the biotransformation of hesperidin and increases in phenolic content and antioxidant activity (Pérez‐Nájera et al. 2018). Moreover, fermentation of orange peel with *Lactiplantibacillus plantarum* for 10 days was also found to be helpful in the reduction of bitterness by 50% associated with the reduction in TFC in a study by Deba‐Rementeria et al. (2023). Although fermentation is cheaper than the use of enzymes, phenolics should be separated from the medium and microbial cells and then purified. In addition, a long time and additional nutrients are required for growth and metabolism of microorganisms in the medium.

## 4 Bioactivity of Phenolics in *Citrus* Peel

Studies to date have shown that individual phenolic compounds or their mixtures extracted from *Citrus* peels possess significant therapeutic properties including antioxidant, antihypertensive, antiobesity, antidiabetic, antiinflammatory, anticarcinogenic, antimicrobial, antithrombogenic, and antiatherogenic activities (Table 3). These bioactivities are mostly attributed to the flavonoids and phenolic acids present in the peels (Oboh and Ademosun 2011; Alu'Datt et al. 2017; Šafranko et al. 2023; Ibrahim, Abdelsalam et al. 2024). *Citrus* flavonoids exert their antioxidant activity by scavenging free radicals, reducing production of reactive oxygen species, chelating metal ions, inhibiting lipid peroxidation, increasing the activity of antioxidant enzymes, and decreasing the activity of oxidizing enzymes in the body (Yoon et al. 2011; De Souza et al. 2016). High level of reactive oxygen species is associated with the pathogenesis of many human illnesses. Zaidun, Thent, and Latiff (2018) reported oxidative stress reducing activities of naringenin associated with a reduction in reactive oxygen species and an increase in the antioxidant activity of superoxide dismutase, catalase, and glutathione that could help in treatment of chronic diseases. Rahib et al. (2024) showed the effectiveness of *C. reticulata* peel extract against abamectin‐induced hepatotoxicity and oxidative injury in rats via its antioxidant, antiinflammatory, and gene‐regulating capabilities. Mulvihill and Huff (2012) reported various bioactivities of *Citrus* flavonoids to improve dyslipidemia, normalize glucose homeostasis, prevent oxidative stress and attenuate inflammation, which can improve metabolic health and reduce cardiovascular disease risk. Desmiaty et al. (2024) showed the antioxidant and antiinflammatory properties of *C. amblycarpa*, *C. aurantiifolia*, and *C. hystrix* peel extracts where naringin and neohesperidin were estimated to be the major effective phenols.

**TABLE 3:** Bioactivity of phenolics present in *Citrus* waste.

*Citrus* species	Phenolic compounds	Bioactivity	Reference
Pummelo (*C. grandis*)	Ferulic acid, vanillic acid, *p*‐coumaric acid, naringenin, diosmin, hesperidin, luteolin, rutin, sinapic acid	Antioxidant, ACE inhibition, α‐glucosidase inhibition	Alu'Datt et al. (2017)
Lemon (*C. limon*)	*p*‐Hydroxybenzoic acid, ferulic acid, vanillic acid, *p*‐coumaric acid, naringenin, diosmin, hesperidin, luteolin, rutin, sinapic acid	Antioxidant, ACE inhibition, α‐glucosidase inhibition	
Grapefruit (*C. paradisi*)	Gallic acid, vanillic acid, chlorogenic acid, *p*‐coumaric acid, naringenin, diosmin, hesperidin, luteolin, quercetin, rutin, sinapic acid	Antioxidant, ACE inhibition, α‐amylase inhibition, α‐glucosidase inhibition	
Shoumati orange	*p*‐Hydroxybenzoic acid, ferulic acid, chlorogenic acid, *p*‐coumaric acid, naringenin, diosmin, hesperidin, rutin, sinapic acid	Antioxidant, ACE inhibition, α‐amylase inhibition, α‐glucosidase inhibition	
Clementine (*C. clementina*)	Gallic acid, ferulic acid, chlorogenic acid, *p*‐coumaric acid, naringenin, diosmin, hesperidin, quercetin, rutin, sinapic acid	Antioxidant, ACE inhibition	
Red blood orange (*C. sinensis*)	*p*‐Hydroxybenzoic acid, ferulic acid, vanillic acid, *p*‐coumaric acid, naringenin, diosmin, hesperidin, rutin, sinapic acid	Antioxidant, ACE inhibition, α‐amylase inhibition, α‐glucosidase inhibition	
*C. latifolia* and *C. sinensis* (peels, pulp waste and seeds)	Hesperidin, hesperetin	ACE inhibition	Ruviaro et al. (2020)
Mandarin peel (*C. reticulata*)	*p*‐Coumaric acid, ferulic acid, rutin, eriocitrin	Antifungal, antibacterial (natural preservative), antioxidant	Liu et al. (2021)
Lemon flavedo (*C. limon*)	Gallic acid, 3,4‐dihydroxybenzoic acid, esculetin, catechin, caffeic acid, epicatechin, vitexin, ethyl 3,4‐dihydroxybenzoate, hesperidin	Antioxidant, α‐amylase inhibition, α‐glucosidase inhibition	Lu et al. (2021)
Brocade oranges (*C. sinensis* L. Osbeck)	Caffeic acid, sinapinic acid, *p*‐coumaric acid, ferulic acid, benzoic acid, *p*‐hydroxybenzoic acid, vanillic acid, syringic acid, didymin, naringin, naringenin, neohesperidin, hesperidin, hesperetin, luteolin, rutin, sinensetin, nobiletin, tangeretin	Antioxidant	Wang et al. (2023)
Lemon peel (*C. limon* Lamas)	Caffeic acid, *p*‐coumaric acid, ferulic acid, *o*‐coumaric acid, rutin, hesperidin, quercetin‐3‐*O*‐glucoside, quercitrin, hesperetin, apigenin	Antioxidant, α‐amylase inhibition, ACE inhibition	Durmus and Kilic‐Akyilmaz (2023)
Sweet orange peel (*C. sinensis*)	8‐hydroxy‐3,4′,5,6,7‐pentamethoxyflavone, 5‐hydroxy‐6,7,8,3′,4′‐pentamethoxyflavone, 5, 6, 7, 8, 4′‐pentamethoxy flavone (tangeretin), 5,6,7,8,3′,4′‐hexamethoxy flavone (nobiletin), 3, 5, 6, 7, 8, 3′, 4’‐Heptamethoxyflavone (3‐methoxynobiletin), 5, 7‐dihydroxy‐4′‐methoxyflavonol, 5, 7, 3′‐trihydroxy‐4′‐methoxyflavonol (tamarixetin), isorhamnetin (3‐methylquercetin), 7‐hydroxy‐3,5‐dimethoxy‐ 3, 4′‐methylenedioxyflavone	Antiinflammatory, anticarcinogenic, antiviral, antioxidant, antithrombogenic, antiatherogenic	Aboul Naser et al. (2020)
Mandarin peel (*C. reticulata*)	Naringin, hesperidin, tangeritin, rutin, D‐limonene, nobiletin, 5,6,7,3′,4′‐pentamethoxyflavanone, 3,5,6,7,8,3′,4′‐heptamethoxyflavone, 6,7,8,3′,4′‐pentamethoxyflavanone, 4H‐pyran‐4‐one, 2, 3‐dihydro‐3, 5‐dihydroxy‐6‐methyl, 2‐methoxy‐4‐vinyl phenol, and n‐hexadecanoic acid, limonoids (nomilol, nomilinate, calamin, isoobacunoate, Ichangin, limonexic acid, oxolimonin)	Anti‐carcinogenic, anti‐inflammatory, wound healing, modulation of bone density, anti‐atherosclerotic, neuropharmacological activities, antiaging potential, antimicrobial, antioxidant	Shorbagi et al. (2022)
Mandarin peel (*C. reticulata* Blanco)	Chlorogenic acid, caffeic acid, ferulic acid, rutin, hesperidin, naringenin, quercetin, hesperetin, tangeretin	Antioxidant, antiproliferative (human breast carcinoma, human colon adenocarcinoma, human liver hepatocellular carcinoma)	Ferreira, Silva, and Nunes (2018)
Lemon waste (flavedo, albedo) (*C. limon* cv. Eureka)	*p*‐Coumaric acid, ferulic acid, sinapic acid, ethyl 3,4‐dihydroxybenzoate, cinnamic acid, vitexin, naringin, naringenin, hesperidin, myricetin	Antioxidant, α‐amylase inhibition, α‐glucosidase inhibition, lipase inhibition	Gavahian, Yang, and Tsai (2022)
Grapefruit	Hesperidin	Antioxidant, pancreatic lipase inhibition	Huang et al. (2020)
Pomelo			
Kumquat			
Mandarin			
Ponkan			
Tangerine			
Lemon			
Sweet orange			
Newhall navel orange (*C. sinensis* L. Osbeck cv. Newhall)	Sinensetin, narirutin, 4′,5,6,7‐tetramethoxyflavone, nobiletin, 3,3′,4′,5,6,7‐hexamethoxyflavone	Antioxidant, antibacterial, tyrosinase inhibition	Guo, Shan et al. (2020)
Bitter orange	Tangeretin, nobiletin	Anticarcinogenic	Lu et al. (2019)
Kumquat (*C. japonica* Thunb)	Gallic acid, protocatechuic acid, p‐hydroxybenzoic acid, chlorogenic acid, caffeic acid, vanillic acid, ferulic acid, sinapic acid, apigenin 7‐glucoside	Antioxidant	Al‐Saman et al. (2019)
Pomelo pulp (*C. grandis* L. Osbeck)	Cigranoside A, B, C, D, E, bergamjuicin, neoeriocitrin, melitidin, rhoifolin, naringin, hesperidin, neohesperidin, isoquercitrin	Antioxidant, α‐amylase inhibition, α‐glucosidase inhibition, pancreatic lipase inhibition	Deng et al. (2022)
Grapefruit pulp (*C. paradisi* Mcfad)	Cigranoside A, B, C, D, E, bergamjuicin, neoeriocitrin, melitidin, rhoifolin, narirutin, naringin, hesperidin, neohesperidin, isoquercitrin		
*C. reticulata* peel	Hesperidin	Antiepileptic	Sharma et al. (2023)
Sweet lime peel (*C. limetta*)	Total phenolics	Antioxidant	Suri et al. (2022)
*C. sinensis* cv. Malta peel	Naringin, phloridzin dihydrate, caffeic acid, catechin, epicatechin, chlorogenic acid, ferulic acid, trimethoxybenzoic acid, coumaric acid	Antioxidant	Nishad, Saha, and Kaur (2019)
Lemon peel	Rutin, astragalin, isomangiferin, naringin, and quercetin	Antioxidant, anti‐fatigue after exercise	Bao, Zhang, and Yang (2020)

Hesperidin as the major flavonoid in peels of different *Citrus* species has attracted attention with its multiple bioactivities including antioxidant, antihypercholesteric, antihypertensive, anticancer, antimicrobial, antiinflammatory, and antidiabetic properties (Samota et al. 2023). In a study by Galati et al. (1994), hesperidin from orange peel was shown to have antiinflammatory and analgesic effects. In another study by Monforte et al. (1995), hypolipidemic activity of hesperidin was demonstrated on rats by decreased cholesterol, low‐density lipoprotein and increased the high‐density lipoprotein levels in plasma. Besides hesperidin, its aglycone hesperetin, naringin, and rutin were shown to improve hyperglycemia and dyslipidemia in diabetic rats (Jayaraman et al. 2018; Fernandes et al. 2010; Mahmoud et al. 2015). De Souza et al. (2016) also reported that supplementing hesperetin decreased atherogenic index and supported antioxidant defense in rats while hesperetin showed a stronger effect on their antioxidant defense system.

Antihypertensive and diuretic effects of hesperidin were shown in studies with normotensive rats and spontaneously hypertensive rats (Galati et al. 1996). Antihypertensive effect of hesperidin was explained by a reduction in ACE‐activity and angiotensin II levels and suppression of oxidative stress markers (Wunpathe et al. 2018). ACE‐inhibitory activity of lemon waste was also related to hesperidin and also hesperetin by Ruviaro et al. (2020). Yousefian et al. (2019) reported that antihypertensive effect of hesperidin was associated with a decrease in the expression of the NADPH oxidase that causes a reduction in the production of reactive oxygen moieties, increase in the bioavailability of nitric oxide and a decrease in the expression of thromboxane‐A2‐synthesis. Razavi et al. (2015) also reported antihypertensive activity of auraptene, a monoterpene coumarin, from *Citrus* species in hypertensive rats.

Antiobesity effect of *Citrus* flavonoids was reviewed by Nakajima, Macedo, and Macedo (2014) and a potential for inhibition of lipid accumulation along with an antiinflammatory effect was revealed. Moreover, they reported that extracts had higher impact on total body weight compared to a single standard compound. In another study, Nakajima et al. (2016) showed that *Citrus* peel extract with high amounts of hesperidin and naringin had inhibitory effect on new adipocyte synthesis and lipid accumulation while its biotransformed form (with more aglycones hesperetin and naringenin) induced lipolysis of fat tissue. In addition, Evans, Sharma, and Guthrie (2012) manifested efficacy of polymethoxylated flavones, tangeretin and nobiletin, on hyperlipidemia and metabolic syndrome where nobiletin had greater bioavailability in in vitro, in vivo and human studies. In vitro and in vivo studies have also indicated a potential antidiabetic activity by *Citrus* flavonoids from peels that was measured as upregulation of insulin secretion, inhibitory activity on α‐amylase and α‐glucosidase, the enzymes involved in postprandial blood glucose elevation and hyperglycemia (Oboh and Ademosun 2012; Alu'Datt et al. 2017; Lu et al. 2021; Lin et al. 2021; Durmus and Kilic‐Akyilmaz 2023).

Several in vitro and in vivo studies have demonstrated that *Citrus* flavonoids have antiinflammatory activity due to inhibition of the synthesis and activities of the proinflammatory mediators including prostaglandin E2, nitric oxide, tumor necrosis factor‐a, and interleukin‐1b (Benavente‐García and Castillo 2008; Khan et al. 2015; Lin et al. 2021). Among *Citrus* flavonoids, naringin, rutin, and tangeretin showed high scores for antiinflammatory activity (Sharma et al. 2022). In addition to the antiinflammatory effect, Tsoupras (2022) manifested antithrombotic effect of phenolic compounds present in the peels of orange cultivars Navalina and Sanguine and mandarin cultivar Clementine. In another study, water and ethyl‐acetate extracts of polyphenols from mandarin (*C. reticulata* Blanco) peel fed to acute colitis‐induced mice reduced colon mucosa damages and the secretion of inflammatory cytokines that proved their possible application in preventing or managing inflammatory bowel diseases (He et al. 2023). Furthermore, a possible bioactivity against Alzhemier disease was reported for *C. maxima* peel extract where 55% inhibitory activity against beta‐secretase (BACE‐1), which is a drug target, was observed (Inthachat et al. 2023). Moreover, silver nanoparticles fabricated with aqueous extract from *C. limetta* peel showed inhibitory effect on cholinesterase related to Alzheimer disease and also on α‐amylase and α‐glucosidase related to diabetes in a dose‐dependent manner in in vitro assays (Sher et al. 2024).

Polymethoxyflavonoids unique to *Citrus* peels are proven to have various bioactivities by in vitro and in vivo studies including antiinflammatory, neuroprotective, anticancer, antiatherogenic, antihyperlipidemic and antidiabetic effects. In a recent study by Lai et al. (2024), an extract from the peel of *C. sinensis* Osbeck cv. Newhall was shown to exert antiglycation, α‐glucosidase and acetylcholinesterase inhibitory activities associated especially with polymethoxyflavones. Li, Fan, and Xi (2019) attributed bioactivity and bioavailability of polymethoxyflavonoids to their high permeability through cell membrane despite their poor aqueous solubility. Zhang et al. (2023) also indicated that high hydrophobicity and low water solubility due to methoxy groups inhibit absorption of polymethoxyflavones; however, their metabolites created by microbial and enzymatic conversions in the large intestine could be effective in their bioactivity. Gao et al. (2019) enriched polymethoxyflavones from the peels of *C. reticulata* by using macroporous resin and showed their hypolipidemic effect on high fat diet‐induced hyperlipidemic mice without toxicity upto 5 g/kg. Nobiletin and tangeretin together with hesperidin in tangerine peel exerted antineuroinflammatory activity in lipopolysaccharide (LPS)‐activated BV2 microglia culture system (Ho and Kuo 20142014). The authors emphasized that lipophilic nobiletin and tangeretin can cross blood–brain barrier and protect neuron cells from oxidative stress‐induced apoptosis. In another study, 5‐demethyltangeretin, an autohydrolysis product of tangeretin, was found to have an anticancer effect in vivo, inhibiting inflammation‐associated skin carcinogenesis (Ma et al. 2014). Tangeretin was also found to have a potential in treatment of drug‐resistant cancer types by acting synergistically in combination with conventional chemotherapeutic agents sensitizing cancer cells (Feng et al. 2016). Wang et al. (2020) also showed that polymethoxyflavonoids especially tangeretin from Ougan (*C. reticulata* cv. Suavissima) extract inhibited gastric cancer tumor growth by inducing cell apoptosis in mice without toxic effects. Not only polymethoxyflavone but also other *Citrus* flavonoids have been shown to possess chemopreventive and chemotherapeutic effects as single agents or as co‐adjuvant for other drugs (Cirmi et al. 2016; Yi et al. 2017; Wang et al. 2020). However, their limited bioavailability and duration in the body are major factors for their action. Combination of flavonoids as in the extracts rather than a single one was recommended as they can tackle cancer from multiple ways (Cirmi et al. 2016). In line with this recommendation, Alamoudi et al. (2022) reported cytotoxicity of aqueous extracts of orange, mandarin and lime peels against human tumor cell lines representing human breast carcinoma.

Antimicrobial effect of *Citrus* polyphenols have been reported in various studies. Pfukwa et al. (2019) showed antimicrobial effect of mandarin peel extract on several pathogens, including *Staphylococcus aureus*, *Escherichia coli*, *Enterococcus faecalis*, *Pseudomonads aeruginosa*, *Listeria monocytogenes*, and *Candida albicans*. El‐Beltagi et al. (2022) determined antibacterial activity of methanol or water extract of orange peel on *S. aureus*, *Bacillus subtilis*, *B. cereus*, or *Klebsiella pneumoniae*. Similarly, Oluwatobi et al. (2024) showed the antimicrobial activity of the extracts of peels, pomace, seeds, and essential oils of lemon and lime against several pathogenic bacteria; however, *B. subtilis* was observed to be resistant to the peel extract. In a study by Genovese et al. (2014), orange and kumquat were found to be the richest sources of phytochemicals like 4′‐geranyloxyferulic acid and boropinic acid which have been discovered as valuable pharmacological agents with anti‐*Helicobacter pylori* activity. Moreover, antiviral and antiinflammatory activities of hesperidin and diosmin have attracted attention for possible treatment of rotavirus and COVID‐19 infections (Akalın et al. 2020). Antibacterial and antifungal effects of mandarin, bitter, and sweet oranges were also determined by Lamine et al. (2021). Ucella‐Filho et al. (2024) reported that antiviral effect of a bio‐oil derived from residual biomass of *C. sinensis* was associated with phenolic compounds, o‐guaiacol being prominent. In addition, lemon and lime peel extracts exhibited antifungal activity against *Aspergillus niger*, *Alternaria*, *Corynespora*, *Fusarium*, and *Rhizopus species* (Oluwatobi et al. 2024).

Nonextractable phenolics have been shown to possess bioactivity comparable to that of the extractable phenolics. ACE‐ and α‐amylase inhibitory activities of the nonextractable fraction of lemon peel obtained by heat, UAE and EAE were found higher than those of the extractable fraction (Durmus and Kilic‐Akyilmaz 2023). Oboh and Ademosun (2011) also reported that bound phenolics fraction of Shaddock peels obtained by base–acid treatments had higher α‐amylase‐ but lower ACE‐inhibitory activity that of free phenolics fraction. On the contrary, activation of ACE and α‐amylase by bound phenolics fraction obtained by acid or alkali hydrolysis of various *Citrus* wastes was observed by Alu'datt et al. (2017). These authors used the edible fruit with albedo and seeds rather than the peel and they had much lower TPC in the bound fraction. Dongre et al. (2024) also demonstrated antidiabetic potential of a hydrodistilled peel residue from *C. sinensis* by inhibiting α‐amylase and α‐glucosidase in a concentration‐dependent manner. In another study, Albuquerque et al. (2019) showed antiinflammatory activity (reducing nitric oxide levels) and prebiotic effect (on *Lactobacillus rhamnosus* GG) of hot water extract of orange by‐products using macrophage and Caco‐2 cell culture, respectively, which was attributed to mostly insoluble‐bound phenolics with a lesser effect of soluble dietary fiber present in the extract. Metabolism of both insoluble‐bound phenolics and dietary fiber by the microflora in colon may exert beneficial effects on the modulation of gut microflora (Rocchetti et al. 2022).

Fermentation or externally added hydrolytic enzymes to *Citrus* wastes can release bound phenolics from cell matrix and/or transform them to their aglycones or small molecular weight metabolites with higher bioactivity. Im, Kim, and Kim (2014) showed that enzymatic hydrolysis of *C. unshiu* peel by cellulase, pectinase and β‐glucosidase had antiproliferative activity and sub‐G1 arrest in human melanoma A375 and colon cancer HCT116 cells at a concentration of 400 μg/mL. In this study, hesperidin, narirutin, and rutin in the extract were hydrolyzed to their aglycones and other phenolics were also released by the enzymes except flavonoids. In another study by Ruviaro et al. (2020), treatment of a *Citrus* by‐product by tannase and β‐glucosidase caused biotransformation of hesperidin to hesperetin and enhanced its antioxidant capacity and ACE inhibitory activity. The extract had equivalent ACE inhibitory activity to that of 1000 μM hesperitin, even though it had less hesperetin content which was explained by possible synergism between different phenolic compounds. Moreover, hesperidin and narirutin enzymatically‐released by pectinases from green yuzu (*C. junos*) have been reported to have antiinflammatory and antiaging effect on skin via the inhibition of matrix metalloprotease‐1 and tyrosinase in addition to antibacterial effect on food‐borne and skin pathogens (Jeong et al. 2004).

Processing conditions can influence the bioactivity of *Citrus* wastes as well. Gavahian, Yang, and Tsai (2022) found an increase in the bioactivity of lemon peels by freeze drying compared to oven drying at 50°C, where 210%, 2%, and 46% increase in inhibition of metabolic enzymes α‐amylase, α‐glucosidase, and lipase was possible, respectively. Ambriz‐Pérez et al. (2021) determined that TPC in free and bound phenolic (obtained by alkali/acid hydrolysis) fractions of Persian lime peel was higher after steam distillation compared to Soxhelet extraction of oil with hexane. However, antioxidant capacity was lower after steam distillation. In the study of Durmus and Kilic‐Akyilmaz (2023), treatment with ultrasound and hydrolytic enzymes instead of heat treatment in extraction of nonextractable phenolics increased antioxidant and α‐amylase inhibitory activity while there was no effect on ACE‐inhibitory activity.

## 5 Bioavailability of Phenolics in *Citrus* Peel

The bioavailability of phenolic compounds affects their physiological activities. The main digestive processes that control the bioavailability of polyphenolic compounds include their release and digestion in the gastrointestinal tract, absorption as aglycones or conjugates, modifications, transportation into the bloodstream and then tissues, and finally excretion (Singh et al. 2020). Solubility, absorption, and biotransformation all have a major role in the bioavailability of any substance. *Citrus* phenolics have low water solubility and oxidation/reduction potential, which contributes to their limited bioavailability, poor stability, delayed digestion, and absorption (Elmeligy et al. 2021). Metabolism and bioavailability of phenolics follow specific pathways that differ widely between individuals and are influenced by a variety of intrinsic (age, gender, gastrointestinal system, gut microbiota, metabolic state, genetic polymorphism), as well as extrinsic (food matrix, co‐consumed food, phenol solubility, dose, and food processing) factors (Visvanathan and Williamson 2022). Absorbed soluble phenolics and their conjugated metabolites are carried by the blood to different organ sites and can exhibit bioactivity.

The bioavailability of phenolic compounds is the percentage of the ingested substance that is absorbed and transported to the systemic circulation and target tissues to exert biological activity (Dima et al. 2020). Phenolic compounds must be first liberated from the food matrix in order to be absorbed. Aglycones may typically be absorbed from the small intestine, however most phenolic compounds are found in foods as esters, glycosides, or polymers that cannot be absorbed in their natural state (Barros and Junior 2019). Food processing, cooking, chewing, and digesting can release phenolics from the food matrix and make them available for absorption in the gut, the degree of which is called bioaccessibility. However, the release of phenolic compounds does not guarantee their complete absorption; only 5%–10% of the ingested soluble phenolic compounds, including those released by the stomach (acid, enzymes) and small intestine, can be absorbed by the small intestine (Cardona et al. 2013). The rate and amount of absorption as well as the kind of metabolites circulating in the plasma are determined by the chemical structure of phenolic compounds rather than their concentration. Moreover, flavanoids must be first hydrolysed into their aglycones before absorption (Ruviaro et al. 2020). The conjugated forms that were not altered in the oral cavity are biotransformed in the gastrointestinal tract through β‐hydrolysis of the sugar moieties in the *O*‐glycoside flavonoids through via phase I (oxidation, reduction, and hydrolysis) and phase II (conjugation) enzymatic detoxification pathways. This results in a variety of water‐soluble conjugate metabolites that can pass the enteric barrier and be distributed to various organs before being excreted in urine (Barros and Junior 2019). Flavonoids with a glucose are deglycosylated and absorbed in the small intestine with the help of β‐glucosidases. If a rhamnose is present, the gut microbiota will deglycosylate the polyphenol rhamnoside (or rutinoside) in the colon (Visvanathan and Williamson 2022). Phenolic compounds have been reported to be more bioavailable when consumed as monoglucosides rather than rutinosides (Nielsen et al. 2006; Visvanathan and Williamson 2022). In addition, phenolic acids are absorbed more in the small intestine compared to flavonols (Zanotti et al. 2015). Moreover, polymethoxylated flavones have a different structure with polymethylation of polyhydroxylated flavonoids than the other flavonoids that increases their oral bioavailability, metabolic stability, and membrane transport in the liver and gut (Evans, Sharma, and Guthrie 2012; Singh et al. 2020).

Hesperidin has limited bioavailability due to the delayed action of the intestinal bacteria necessary to liberate the rutinose moiety of hesperidin before absorption of its aglycone hesperetin. Hesperetin‐7‐glucoside, which is produced by enzymatically removing the rhamnose sugar from hesperidin, had a three‐fold higher bioavailability in human subjects (Habauzit et al. 2009; Nielsen et al. 2006). Lee et al. (2012) also reported that the enzymatic conversion of hesperidin to hesperetin‐7‐O‐glucoside resulted in increased solubility and bioavailability. Moreover, hesperidin was reported to have low membrane permeability compared to its aglycone and be absorbed mostly via the paracellular route (Wdowiak et al. 2022). Londoño‐Londoño et al. (2010) also stated that hesperetin with acyl chains and a more planar shape has a greater interaction with membranes than hesperidin which is positioned at the level of the polar head because of its rutinoside moiety. Better interaction of aglycones with membranes can explain their high bioavailability.

After the ingestion of flavonoids, conjugated metabolites of *Citrus* flavonoids have been found in human plasma. Miyake et al. (2006) identified glucuro‐ and/or sulfo‐conjugates of eriodictyol, homoeriodictyol, and hesperetin in human plasma after ingestion of flavonoids, eriocitrin and hesperidin, and observed that the aglycones were absorbed faster and at higher concentrations than flavonoids. Ameer et al. (1996) reported a bioavailability of around 25% for naringin and hesperidin which were glucuronidated after absorption before being excreted in the urine. Wang et al. (2006) also demonstrated the sulfate and glucuronate conjugates of naringenin in the plasma of rats after consumption of naringin. Guo et al. (2020) determined that naringin sulfates were absorbed by the ileum and cecum, deglycosylated metabolites by the duodenum and cecum, and phenolic acids by the ileum, cecum, and colon. In addition, they reported that hesperidin sulfates may be absorbed by the duodenum and ileum, whereas metabolites of naringin or hesperidin were not absorbed by the jejunum.

The bioavailability of phenolic compounds may also be impacted by the food matrix with proteins, lipids, and polysaccharides surrounding them in the digestive system. Food matrix generally delays and extends the absorption of phenolics by hindering chemical and enzymatic reactions involved in the metabolism of phenolics (Visvanathan and Williamson 2022). Pereira‐Caro et al. (2022) showed that the amount of flavanone metabolites recovered in urine was dramatically decreased by 3‐fold when orange juice and oat β‐glucan were consumed together. This finding was attributed to the increase in the viscosity of chyme preventing absorption in the stomach. Kruger, Sus, and Frank (2020) used an *in vitro* gastrointestinal digestion model together with Caco‐2 cells to examine the impact of pectin and sucrose on the bioaccessibility of naringenin. Bioaccessibility of naringenin was reduced by 65% when 2% pectin was present; however, the amount of bioaccessible naringenin that was absorbed by Caco‐2 cells increased from 47% to 95% when pectin was present. Encapsulation of naringenin by pectin was claimed to cause the increase in its absorption. Additionally, adding 5% sucrose to naringenin at a concentration of 700 μM with 2% pectin decreased the inhibitory impact of the pectin and raised naringenin bioaccessibility from 8% to 15%. Moreover, Jakobek (2015) found that the interaction between lipids and polyphenols can reduce lipase activity and fat absorption while polyphenols can be protected by lipids improving their transportation and activity in the gastrointestinal tract.

Metabolism of nonextractable phenolics differ from that of the extractable phenolics of which polymeric structure and chemical bonds between food matrix components and phenolics reduce their solubility and bioavailability. Insoluble‐bound phenolics, along with other unabsorbed phenolics by stomach and intestine, are transmitted to the colon intact with other insoluble food components. There, they can have beneficial health effects through their impact on colonic microflora in addition to being catabolized by them to absorbable low molecular weight metabolites (e.g., phenylacetic, phenylpropionic, urolithine) or nonabsorbable metabolites acting as antioxidants *in situ* (Wdowiak et al. 2022; González‐Sarrías, Espín, and Tomás‐Barberán 2017; Arranz, Silván, and Saura‐Calixto 2010). The microbial flora of colon can hydrolyze glycosidic bonds and break down insoluble phenolics into smaller phenolic compounds (Arranz, Silván, and Saura‐Calixto 2010). In addition, a prebiotic‐like effect of unabsorbed phenolics and their metabolites in the colon have been reported (Espín, González‐Sarrías, and Tomás‐Barberán 2017). Low rate of metabolism of insoluble‐bound phenolics and their persistance in the plasma for 3–4 days have been suggested as a potential for exerting their bioactivity (Zhang et al. 2020). Absorbable low molecular weight metabolites of nonextractable phenolics have been shown to remain in circulation for a longer time than extractable phenolics and exert antiinflammatory and antioxidant effects in in vivo studies (González‐Sarrías, Espín, and Tomás‐Barberán 2017). Hesperidin has been shown to be hydrolysed by gut microbiota to hesperetin and its phase II conjugates that are found in the circulation (Vallejo et al. 2010). These metabolites of hesperetin can exhibit antiinflammatory and antioxidant effect in human body as shown in in vivo studies (González‐Sarrías, Espín, and Tomás‐Barberán 2017). Overall, insoluble‐bound phenolics can exert health benefits by their antioxidant effect in situ and through their metabolites produced by gut microbiota and their effects on gut microbial population.

Common food processing techniques applied to wastes can affect phenolic content, bioactivity and bioavailability. The processing method and parameters (medium, intensity, and duration) used have impacts on the properties of the resulting product. Rodríguez‐Roque et al. (2015) investigated the effect of thermal treatment at 90°C for 60 s on the bioaccessibility of phenolics in fruit juice and found that the bioaccessibility of phenolic acids decreased by 12.7%, that of flavonoids increased by 2.65% and that of TPC decreased by 4.17% while PEF and HHP had no effect. Castello et al. (2020) observed faster absorption but similar bioavailability of phenolic metabolites of orange juice before and after alcoholic fermentation in human subjects which was explained by the effect of fermentation on the pulp content, juice cloud particle size and moderate alcohol content. In a study with an in vitro digestion model, Gil‐Izquierdo et al. (2003) demostrated that while freezing and cold storage significantly reduced the solubility and permeability of the flavanones in orange juice, pasteurization and concentration had no effect on either. Ozdemirli and Kamiloglu (2024) investigated in vitro gastrointestinal digestion stability of polyphenols from orange and lemon peels after industrial blanching, cutting, and freezing treatments. Blanching resulted in an increase in the levels of major flavonoids after in vitro gastrointestinal digestion while cutting after blanching reduced them. There was no effect of blanching‐cutting on the content of phenolic acids after gastrointestinal digestion. While freezing treatment slightly reduced bioaccessibility of flavonoids and phenolic acids in the case of orange peel, that of polyphenols from lemon peel was enhanced.

Encapsulation of polyphenols protect them from outer environment and during gastrointestinal metabolism. This can also provide a solution for their poor water solubility that limits their bioavailability. Colloidal delivery systems with pectin, sodium alginate, cellulose, maltodextrins, cyclodextrins and whey proteins have been found effective in enhancement of stability, dispersibility, and bioaccessibility of encapsulated polyphenols from *Citrus* pomace (Caballero et al. 2022). García‐Martínez, Camacho, and Martínez‐Navarrete (2023) encapsulated orange peel extract with gum Arabic and esterified corn starch with octenyl succinic groups by freeze drying. Stability of hydrophilic compounds were improved in the presence of biopolymers during freeze‐drying especially for higher biopolymer concentrations. Bioaccessibility of hesperidin and narirutin slightly increased by gum Arabic and esterified starch. Galindo et al. (2022) also encapsulated orange juice waste by incorporation of gum Arabic and bamboo fiber for the stabilization of ascorbic acid and hesperidin. In another study, bioaccessibility of lipophilic polymethoxyflavones, tangeretin and nobiletin, was shown to be increased by 5‐ and 2‐fold, respectively, by loading into high internal phase emulsions stabilized by whey protein isolate and low methoxyl pectin in a dynamic in vitro digestion model (Wijaya et al. 2020). Rosales and Fabi (2023) suggested nanoencapsulation of phenolic compounds from wastes to enhance their stability and bioavailability by using natural polysaccharides such as pectin. In this line, Mariano et al. (2024) encapsulated orange‐derived hesperetin in zein/pectin nanoparticles and found that encapsulation significantly increased its bioaccessibility (~560%) compared to that of dispersed in deionized water (11.5%). Nonetheless, safety of these nanoparticles needs to be determined in in vitro and in vivo toxicity tests for long‐term consumption.

## 6 Application of Bioactive Phenolics in Foods

With the increased awareness of consumers about the connection between diet and health, the global food consumption trend is shifted towards functional foods. Consequently, extensive research is being conducted to investigate the foods supplemented with phytochemicals to improve health. In line with this trend, the exploration of *Citrus* peel as a potential source of phenolic compounds has gained significant attention with a focus on its utilization in various industries such as food, cosmetic and pharmaceutical (Mohsin et al. 2021; Wedamulla et al. 2022; Martínez‐Zamora et al. 2023).

The applications of *Citrus* peel in the food products are mainly based on their antioxidant and antimicrobial activities as well as nutrient content. Fortification with powdered peel or extracts therefrom is common techniques to enhance stability, nutritional value and functionality of the food products (Kaur, Panesar, and Chopra 2023a). Polyphenolic compounds present in *Citrus* wastes can serve as potent lipid oxidation inhibitors in meat and fish products improving oxidative stability and prolonging their shelf life (Farag et al. 2020). Nishad et al. (2018) shown that the lipid and protein oxidations in frozen meat during storage were reduced by the addition of grapefruit peel. *Citrus* peel extract was also found effective in inhibiting the formation of heterocyclic amines and advanced glycation end products in grilled pork meat patties (Xu, Zhao et al. 2023). Shelf life of fats and oils can be extended by adding extracts from *Citrus* peel. Interestingly, orange peel extract exhibited a higher inhibitory effect on oxidation of sunflower and soybean oils than synthetic antioxidants (Shehata et al. 2021). Moreover, the addition of a nano‐emulsion of grapefruit peel phenolics to mustard oil was shown to extend its oxidative stability (Nishad et al. 2021).

*Citrus* peels have been incorporated into confectionery and bakery products due to their high contents of dietary fiber and bioactive compounds. For example, lime and lemon peels added savory cake exhibited higher ascorbic acid, β‐carotene and phenolic contents and free radical scavenging activity along with acceptable sensory properties (Das and Gupta 2018). In the same line, El‐Beltagi et al. (2022) produced an acceptable sponge cake by substituting wheat flour with 10% orange peel powder with high antioxidant activity. Enrichment of biscuits was possible with lemon peel and pomace, kinnow peel and pulp residue and orange peel which enhanced oxidative stability and fiber content (Imeneo et al. 2021; Purewal, Kaur, and Sandhu 2023; Obafaye and Omoba 2018). Similarly, addition of sweet orange by‐products into wheat bread and cookies incorporated phenolics as well as minerals and fiber (Castro et al. 2020). Gómez‐Mejía et al. (2023) also added mandarin peel extract into wheat bread for increasing antioxidant potential. Use of bitter orange albedo along with purple potato in sourdough bread extended mold‐free shelf life; however, bitterness was perceptible at the level of 0.75% used (Taglieri et al. 2021). To overcome the bitterness problem, Singla et al. (2021) treated kinnow pomace with naringinase and used it to enrich pasta with antioxidants. Asif et al. (2023) reported that *Citrus* pomace addition significantly increased the antioxidant activity and fiber content of corn extrudates with acceptable sensory properties at a level of 5%. Another study conducted by Rathod and Annapure (2017) showed that blend of lentil and orange peel can be good alternative for extruded snacks with great nutritional value.

Orange, mandarin and lime peel powders were used for fortification of yoghurt. Increase in number of probiotics and acceptable sensory properties were achieved at a level of 1% or 3% addition (Alamoudi et al. 2022). Similarly, Fathy et al. (2022) showed that addition of 0.5% lemon or orange peel powder enhanced probiotic viability for *Lactobacillus acidophilus* and *Bifidobacterium* spp. and antimicrobial activity against *S. aureus*, *B. subtilis*, and *E. coli* without affecting overall acceptability in yoghurt. According to a study by Kandyliari et al. (2023), antioxidant activity of kefir was intensified when fortified with bitter orange or lemon peels compared to control. In a study from a different perspective, Guzmán et al. (2024) added orange pulp to the diet of goats and showed that the cheese made from the milk of these animals had increased vitamin E content, TPC, and antioxidant activity.

*Citrus* peel extracts have been explored for developing active food packaging material to extend shelf life of various food products by their antioxidant and antimicrobial effects (Yadav et al. 2023). Santos et al. (2023) developed bioplastic films from orange peel by a hydrothermal process where all films had high antioxidant and UV absorption capacities being usable as active packaging materials for oxidizable products. Jridi et al. (2019) used blood orange peel extract along with fish skin gelatin in an edible film formulation. Usage of lemon peel extract at a level of 20% in an active starch‐based packaging material allowed reductions in moisture loss, peroxide value and total volatile basic nitrogen and preserved the fatty acid profile of fresh smoked fish during storage (Oluwasina and Awonyemi 2021). Jodhani and Nataraj (2021) showed that lemon peel extract in an edible aloe gel coating extended the shelf life and reduced the quality loss in banana. In another study, mosambi peel extract fortification of surimi improved gelling properties and overall acceptability (Sharma et al. 2023). Moreover, peel powder of *C. unshiu* was used at a concentration of 1%–7% in the development of a functional jelly candy (Baek, Ryu, and Paik 2023). Aiello et al. (2024) also obtained functionalized gelatin ingredients by grafting polyphenols from peel extracts of orange and lemons to gelatin and produced functional gummies from these.

Polyphenols in *Citrus* peels and their extracts exert antimicrobial activity through changes in the membrane integrity and metabolism of microbial cells. Khalil, Sharaby, and Abdelrahim (2023) developed a grapefruit pectin‐based edible film incorporated with an extract from grapefruit or lemon peel that was effective against *E. coli* strains on tomatoes during storage. Extracts from sweet orange, lemon, tangerine and grapefruit peels were shown to inhibit growth of food‐borne Gram‐positive and Gram‐negative bacteria as well as fungi (Papoutsis et al. 2018b; Shehata et al. 2021; El‐Beltagi et al. 2022). A bioelastomer fabricated using mandarin peel extract and polydimethylsiloxane also exhibited antimicrobial effect on food‐borne Gram‐positive and Gram‐negative bacteria (Lee et al. 2023). Zappia et al. (2023) reported that the application of alginate‐based edible coating with an extract from lemon by‐products including peel on minimally processed radish (*Raphanus sativus* L.) not only reduced mesophilic aerobic counts but also delayed respiration extending the shelf life. In another study, microbial load of butter was reduced by treatment with sweet lime peel powder (Maqbool et al. 2023).

Aqueous extracts of orange, lemon and citron peels were found effective in inhibition of the growth and biofilm formation of pathogenic *Staphylococcus* and *Pseudomonas* strains (Caputo et al. 2018). Andrade et al. (2023) also showed that polyethylene‐based packaging with 4% lemon extract and two polylactic‐based packaging materials with 4% and 6% lemon extract delayed lipid oxidation in almonds and inhibited the growth of microorganisms and lipid oxidation in raw beef. Additionally, application of composite coating films with bitter orange peel extract on raw chicken fillets decreased the microbial growth, free fatty acids, peroxide value and hydroperoxide generation (Azizkhani, Kavosi, and Partovi 2023). In a study by Gopalakrishnan et al. (2023), delaying yeast and mold growth in bread was possible by wrapping with a cellulose film incorporated with kinnow peel extract.

Aforementioned studies have proven that polyphenols from *Citrus* peels are promising natural food ingredients; however, there are some limitations like instability, low water solubility, low bioavailability and bitterness (naringenin) to extend their usage of in food industry (Wedamulla et al. 2022; Caballero et al. 2022). Thus, encapsulation has been identified as one of the techniques to overcome these limitations. Spinelli et al. (2018) reported higher bioaccessibility for polyphenols of sweet orange epicarp extract in fish burgers when added as microcapsules. In another study, encapsulated phenolic extract from lime waste and hesperidin were used to enrich orange juice without bitterness and destruction during heat treatment (Afkhami, Goli, and Keramat 2018). Encapsulation was found succesfull in reducing bitterness and protecting phenolics where 80% of hesperidin was destroyed by heat treatment without encapsulation.

Bioactive phenolics from *Citrus* peel can also find applications in the pharmaceutical sector. Nonfood uses of *Citrus* peel also encompass its utilization as a substrate for producing bio‐adsorbents, bio‐fuels, bio‐fertilizers, packaging materials and activated carbon (Sharma et al. 2017). Phytochemicals obtained from *Citrus* peel are also utilized in cosmetic formulations designed for skin, hair, and nail care. They are included in formulation of antifungal and antibacterial lotions, soaps, perfumes and toiletries (Mahato et al. 2018). The antioxidants found in *Citrus* peel play a role in delaying skin aging, mitigating oxidative damage and healing various skin‐related concerns such as acne, wrinkles, and dark spots (Sharma et al. 2017). A study by Apraj and Pandita (2016) showed that *C. reticula* peel extract can be effectively employed in anti‐wrinkle skincare formulations due to its antioxidant and anti‐enzyme attributes. Furthermore, another study demonstrated that orange peel extract exhibited significant anti‐tyrosinase activity, making it a promising ingredient for whitening cream formulations (Wuttisin et al. 2017). A recent study also showed that lemon peel extract incorporated into nanoemulgel formulations can be used as nontoxic, antimicrobial and alcohol‐free hand sanitizer which could be an alternative to the commercial alcohol‐based formulations (Ibrahim, Shalaby et al. 2024). Moreover, the presence of essential oils and a unique fragrance of *Citrus* peel further supports its suitability for incorporation into cosmetic products (Pinto et al. 2021).

*Citrus* peels have a large potential for upcycling and reaching a zero‐waste target by their rich composition of nutrients and bioactive components. Phenolic compounds are one of the major components along with pectin, essential oil, ascorbic acid and dietary fiber (Suri et al. 2022; Xu, Li et al. 2023; Magalhães et al. 2023). Integrative biorefinery processes can be developed for step‐wise extraction of these components and manufacture of value‐added ingredients and nutraceuticals. Use of green solvents and technologies in these processes will also ensure achieving sustainability goals in the food industry (Yadav et al. 2022). On the other hand, *Citrus* peels can be utilized directly as functional food ingredients or nutraceuticals as a source of multiple health‐improving components. However, there is a possible risk of contamination of the end products by pesticides on the surface of the peels which needs to be considered in their valorization. Nevertheless, a study by Otero et al. (2023) evaluated the safety of ascorbic acid recovered from *Citrus* peels and concluded that it was safe for use.

## 7 Conclusion

*Citrus* peel waste stands out with a wide variety of phenolics with numerous health‐improving bioactivities including antioxidant, antihypertensive, antihyperlipidemic, antidiabetic, antiinflammatory, anticarcinogenic, antimicrobial, antithrombogenic, and antiatherogenic activities that they possess. Both extractable and nonextractable phenolics are present at significant amounts in the peel of different *Citrus* species that have a potential to be valorized. Slow absorption of insoluble nonextractable phenolics and their sustained release into the blood stream can enhance their bioactivity. The fact that insoluble phenolics reach colon intact and are metabolized by the colonic microflora results in modulation of microbial flora in turn, in addition to the production of new absorbable metabolites, provides additional health benefits to *Citrus* peels. Moreover, the presence of ascorbic acid and other antioxidants and dietary fiber in *Citrus* peel enhance their health effects. Especially, interactions between dietary fiber and insoluble phenolics in the colon can positively modify metabolism of phenolics and beneficial effects of both on microbial flora. Although the extractable phenolics from *Citrus* peels were characterized in terms of bioactivity and bioavailability in vitro and in vivo studies, there is a scarcity of information especially in vivo and clinical studies on bioavailability, bioactivity, and health effects of the nonextractable phenolics.

Polyphenols found in *Citrus* peel can be extracted by green technologies for use in a circular economy scheme. Although green extraction methods with less solvent, energy and time consumption such as ultrasound, microwave, high pressure and SCF extraction have been investigated in the experimental studies, scale‐up of these processes are required for industrial production. Stability of phenolics must be ensured in the process or by an additional encapsulation process to improve their bioactivity and bioavailability in the end product.

*Citrus* peels or phenolic extracts therefrom can be upcycled as antioxidant or functional natural food ingredients. In addition, incorporation of *Citrus* phenolic extracts as natural antioxidant and antimicrobial agents to edible or packaging film for improving quality and extending shelf life of food products is a promising application. Moreover, new food supplements or pharmaceuticals can be produced from the extracts towards improvement of health status of individuals in need. However, stability, toxicity, bioactivity, and bioavailability of the phenolics need to be determined by more in vivo and clinical studies before their use as food supplements or pharmaceutical preparations.

## Author Contributions

**Nihal Durmus:** conceptualization, visualization, writing – original draft. **Zehra Gulsunoglu‐Konuskan:** conceptualization, visualization, writing – original draft. **Meral Kilic‐Akyilmaz:** conceptualization, supervision, methodology, writing – review and editing.

## Conflicts of Interest

The authors declare no conflicts of interest.

## Data Availability

The authors have nothing to report.
